# The ERAS nursing care strategy for patients undergoing transsphenoidal endoscopic pituitary tumor resection: A randomized blinded controlled trial

**DOI:** 10.1515/med-2025-1139

**Published:** 2025-03-03

**Authors:** Min Tang, Seidu A. Richard, Chaofeng Fan, Zhen Luo, Wei Zhu, Qian He, Zhigang Lan, Lijuan Duan

**Affiliations:** Department of Nursing, West China Hospital, Sichuan University, Chengdu, Sichuan, 610041, P.R. China; Department of Neurosurgery, West China Hospital, Sichuan University, Chengdu, Sichuan, 610041, P.R. China; Institute of Neuroscience, Third Affiliated Hospital, Zhengzhou University, Zhengzhou, 450052, China; Department of Biochemistry and Forensic Sciences, School of Chemical and Biochemical Sciences, C. K. Tedam University of Technology and Applied Sciences (CKT-UTAS), Navrongo, Ghana; Department of Nursing, West China Hospital, Sichuan University, 37 Guo Xue Xiang Road, Chengdu, Sichuan, 610041, P.R. China; Department of Neurosurgery, West China Hospital, Sichuan University, 37 Guo Xue Xiang Road, Chengdu, Sichuan, 610041, P.R. China

**Keywords:** ERAS, LOS, nursing, TEP, self-care, food intake

## Abstract

**Introduction:**

Transsphenoidal endoscopic pituitary (TEP) tumor resection is performed through the nose via the sphenoid sinus to remove tumors from the pituitary gland. Also, enhanced recovery after surgery (ERAS) was adapted to reduce physical and physiological traumatic stress response of surgical patients.

**Methods:**

A total of 174 patients who underwent TEP tumor resection in our department from August 2021 to June 2022 were randomly divided into non-ERAS group and ERAS group. The main primary observational indicator was postoperative self-care ability parameters such as early urethral catheters’ removal and postoperative food intake. Also, secondary indicators such as postoperative complications, average length of hospital stay (LOS), and total hospital cost were compared.

**Results:**

The overall self-care ability of the ERAS group was higher than that of the non-ERAS group 24 h after surgery (35 points vs 20 points, *p* < 0.001). Also, food intake on the first day after surgery was higher than that of the non-ERAS group (*p* < 0.001). Furthermore, the average LOS in the ERAS group was lower than that of the non-ERAS group (4 days vs 7 days, *p* < 0.01). Additionally, the average hospitalization cost in the ERAS group was lower than that of the non-ERAS group (32, 886 RMB vs 48, 125 RMB, *p* < 0.001).

**Conclusion:**

ERAS nursing strategy promoted early recovery of self-care, shorten the average LOS, and reduce hospitalization costs without increasing the incidence of postoperative complications.

## 1 Introduction

Transsphenoidal endoscopic pituitary (TEP) tumor resection is performed through the nose via the sphenoid sinus to remove tumors from the pituitary gland and skull base [1]. Notably, TEP approach fully exposes the lesion site, expand the surgical field, and increase the probability of complete resection of the lesion [2]. Also, it has the advantages of short operation time, small wound, and less bleeding [2]. Furthermore, TEP approach has been shown to improve tumor resection rates and reduce the incidence of surgery-related complications such as diabetes insipidus, cerebrospinal fluid (CSF) rhinorrhea, as well as nasal and sphenoid sinus injury [1]. Thus, perioperative nursing strategies need to be optimized urgently to reduce the physiological and psychological stress of patients during the perioperative period, reduce perioperative discomfort, reduce complications, and accelerate patients’ recovery.

Enhanced recovery after surgery (ERAS) is an evidence-based medical care which is aims at reducing the physical and physiological traumatic stress response of surgical patients [3–5]. It is a multidisciplinary approach and involves diverse group of healthcare professionals who work together in an interconnected as well as collaborative manner with confidence in sharing expertise, knowledge, as well as skills to optimize the patient’s entire hospital pathway [6]. The team often includes pre-admission staff, nurses, dieticians, physiotherapists, occupational therapists, social workers, as well as doctors [6].

In the ERAS, patient’s management involves the whole process of hospitalization, such as preoperative, intraoperation, postoperative, as well as post-discharge [4]. Interestingly, the ERAS strategies are helpful in the improvement of perioperative safety and satisfaction, reduce postoperative hospital stay as well as reduce patient healthcare costs, decrease postoperative complication, and readmission rates in protocol for patient’s undergoing TEP tumor resection [7], however, not in clinical trials in patient’s undergoing TEP tumor resection.

In this study, we compared the ERAS nursing strategy to traditional method of nursing patient undergoing TEP tumor resection. We speculated that the ERAS nursing strategy could accelerate recovery. It could shorten the duration of fasting and drinking in the preoperative period of pituitary tumors. Also, this strategy could enhance patient’s self-care ability such as urine control after early urethral catheters’ removal, postoperative food intake, decrease postoperative complications, average length of hospital stays (LOSs), as well as total hospital cost following TEP tumor resection. The above parameters will provide clear clinical guidelines for effective and efficient nursing patients undergoing TEP tumor resection.

## 2 Materials and methods

### 2.1 Study design and patient population

The study was a randomized blinded controlled trial designed in parallel. Patients who underwent TEP tumor resection from August 2021 to June 2022 at the Department of Neurosurgery in West China Hospital of Sichuan University were randomly assigned into ERAS group and non-ERAS group in this clinical trial. General information such as age and gender were collected at the time of admission. Definitive diagnosis such as the type of tumor was established during the operation via frozen sections and confirmation done via histopathological analysis.

The main primary observational indicator was postoperative self-care ability parameters such as urine control after early urethral catheters’ removal and postoperative food intake. Also, secondary indicators such as postoperative complications, average LOS, and total hospital cost were compared. Patient inclusion criteria were (1) patients with pituitary tumor finally diagnosed after magnetic resonance imaging (MRI)/computed tomographic (CT) and hormone examination, (2) tumor size below 3 cm × 3 cm, (3) surgical plan: TEP tumor resection, (4) age ≤60 years old, and (5) agree to participate in this study. Exclusion criteria included: (1) patients with other malignant tumors and serious infections, (2) patients with severe organ dysfunction, (3) patients who are unable to communicate normally, and (4) the patient is disabled or dies due to reasons not related to the disease or accident.

### 2.2 Sample size and randomization

Targeting a power (*p*-value) <0.05 and probability = 0.90, the sample size in the ERAS group = 76 and non-ERAS group = 79 were calculated using the PASS 25 software (https://www.ncss.com/software/pass/). Additionally, assuming that the loss to follow-up rate of the study subjects is 10%, the sample size in ERAS group = 76 ÷ 0.9 = 84 cases and control group = 79 ÷ 0.9 = 87 cases. In the end, 86 subjects were included in the ERAS group and 88 patients in the non-ERAS group. Also, the randomization was a blinded controlled trial designed in parallel. Using a computer-based Excel software, randomization codes were generated for both the ERAS group as well as non-ERAS group and held in sealed opaque envelopes by lead investigator. Patients were asked to pick from collection of sealed opaque envelopes by a train nurse who assigned them to various groups such as ERAS group and non-ERAS group at random. Thus, patients were blinded to interventions while the nurses were divided in ERAS group and non-ERAS group. There were no changes to trial outcomes after the trial commenced.

### 2.3 ERAS nursing protocol

A comparison of nursing protocol for ERAS and non-ERAS for undergoing TEP tumor resection is as shown in Table 1.

**Table 1:** Comparison of nursing protocol for ERAS and non-ERAS for undergoing TEP tumor resection

Strategy	ERAS group	Non-ERAS group
**Preoperative aspects**		
Patient education	All-inclusively patient education	Conversional/routine education
	Benefits of ERAS management	Benefits of non-ERAS management
	Documentation of basic amount of food eaten by the patients before their operations	Conversional/routine eating prior to surgery
	Patients and their relatives were given information leaflets on the ERAS management	Conversional/routine education
Preoperative fasting	Fasting for 6 h prior to surgery	Fasting for 6 h prior to surgery
**Perioperative and early postoperative aspects**		
Anesthetic and analgesic regimens	Sedation, analgesia, and IV fluids were given according to the doctor’s instructions	Sedation, analgesia, and IV fluids were given according to the doctor’s instructions
	After surgery, awake patients were guided as to how to mobilize in bed after general anesthesia	After surgery, awake patients were guided as to how to mobilize in bed after general anesthesia
ECG monitors and oxygen inhalation	The ECG monitoring and oxygen inhalation were stopped when the patient recovered fully from general anesthesia	ECG monitors and oxygen inhalation were stopped on the third day after surgery according to the doctor’s instructions
Patient mobilization and early initiation of food intake	Perioperative and early postoperative accelerated rehabilitation nursing education which included strengthening psychological nursing, accelerated rehabilitation nursing modalities and their significance as well as seek total cooperation of patients and their relatives or families	The patients to get out of bed and move about after ECG monitors were removed
	The patients’ head was supported with pillows in bed and during sleep, and the heads of the beds were raised to about 15–30°	Conversional management
	The patients were instructed to carry out active limb movements in bed, such as flexion and straightening of both lower limbs	Conversional management
	If there was no nausea or vomiting 1 h after recovering from anesthesia, a small amount of warm water was given intermittently as well as taking 200 mL of carbohydrate nutrition powder with water 2 h in advance of the operation	Conversional management
	If there was no nausea or vomiting within 6 h, adequate amount of water was given	Conversional management
	If the patient did not experience any discomfort, a liquid diet was started, and a normal diet was given on the second day after the surgery	Conversional management
Urinary catheters	The urethral catheters were removed under general anesthesia in the anesthesia recovery room before the patient recovered from anesthesia	The urinary catheters were removed on the third day after surgery as instructed by the doctor based on the patient ability to get out of bed to urinate
**Late postoperative aspect**		
Postoperative food intake	The late postoperative care was same in both groups	
Postoperative complications		
Overall postoperative self-care ability		
Discharge guidance		
Hospitalization expenses		

### 2.4 Preoperative aspects

The non-ERAS group was given routine preoperative education. However, the patient education was all-inclusively carried out by a nurse who is trained to do so when the decision to operate on the patient was made at the clinic in the ERAS group. The education parameters included age as well as comorbidities and their upkeep at home. Their relatives or care providers were also properly educated on the nature of their illness and kind of support and concerns they ought to provide. Also, in the ERAS group, the benefits of this kind of management were discussed.

The perioperative as well as the postoperative course of the management was highlighted to the patients and their relatives. Specific deliberating signs were highlighted and patient’s relative was asked to contact the front desk nurse immediately when such signs are observed or reported by the patients. The patient relatives were also with the patients throughout perioperative and the postoperative period as per our hospital policy. The basic amount of food eaten by the patients before their operations was documented in both the groups by a dietician. Furthermore, patient information leaflets were also given to the patients and their relatives [7]. Preoperative diet management regime consisted of fasting for 6 h prior to surgery in both groups.

### 2.5 Perioperative and early postoperative aspects

The non-ERAS group was given routine perioperative care. Standardized anesthetic and analgesic regimens were implemented. Sedation, analgesia, and intravenous fluids were given according to the doctor’s instructions, and after surgery, awake patients were guided as to how to mobilize in bed after general anesthesia. The electrocardiogram (ECG) monitors for oxygen inhalation were stopped on the third day after surgery according to the doctor’s instructions to assist the patients to get out of bed and move about. Also, the urinary catheters were removed on the third day after surgery as instructed by the doctor based on the patient’s ability to get out of bed to urinate.

In the ERAS group, implementation of the ERAS nursing strategy was carried out for standardized anesthetic and analgesic regimens just as the non-ERAS. Furthermore, in the ERAS group early postoperative activities were initiated. The ECG monitoring and oxygen inhalation were stopped when the patient recovered fully from general anesthesia. The doctors and nursing integration team implemented the ERAS nursing strategies such as perioperative accelerated rehabilitation nursing education which included strengthening psychological nursing, accelerated rehabilitation nursing modalities and their significance as well as seek total cooperation of patients and their relatives or families [8].

In the ERAS group, the patients’ head was supported with pillows in the bed and during sleep, and the heads of the beds were raised to about 15–30°. Also, the patients were instructed to carry out active limb movements in the bed, such as flexion and straightening of both lower limbs. If there was no nausea or vomiting 1 h after recovering from anesthesia, a small amount of warm water was given intermittently as well as taking 200 mL of carbohydrate nutrition powder with water 2 h in advance of the operation. However, if there was no nausea or vomiting within 6 h, adequate amount of water was given. Also, if the patient did not experience any discomfort, a liquid diet was started, and a normal diet was given on the second day after the surgery. Furthermore, in the ERAS group, the urethral catheters were removed under general anesthesia in the anesthesia recovery room before the patient recovered from anesthesia.

### 2.6 Late postoperative aspect

The late postoperative care was same in both groups. The amount of food eaten by the patient after surgery was calculated according to the basic amount of food eaten by the patient before the operation on the first day after surgery. The assessments grades include, no food at all, 1/3, 1/2, 2/3 of the preoperative food intake, as well as complete preoperative food intake. Postoperative complications include diabetes insipidus, CSF leakage, intracranial infection, electrolyte imbalance, as well as visual impairment. The assessment criteria for diabetes insipidus included 24 h urine output >40 mL/kg in adults, accompanied by urine specific gravity <1.005, urine osmolality 50–200 mOsm/(kg H_2_O), and urine as pale as water.

Also, the patient was considered to have CSF rhinorrhea if there was a flow of intermittent or continuous clear watery fluid from the patient’s nasal cavity. Furthermore, the assessment criteria for intracranial infection included the patient with clinical symptoms such as high fever, headache, vomiting, and positive meningeal irritation, as well as growth of bacteria in patients CSF cultures. However, the assessment criteria for electrolyte disorder were gross anomalies in electrolyte parameters such as potassium, sodium, chloride, magnesium, as well as plasma osmolality and acid–base imbalances. Impaired vision was assessed via visual field examination to establish vision loss or visual field changes by an ophthalmologist.

### 2.7 Overall postoperative self-care ability, discharge guidance, and hospitalization expenses

The Chinese version of the Barthel index (BI) rating scale which has a total of 100 points was used to assess the overall patient’s self-care ability in both groups. The BI is a scale that measures a patient’s ability to perform activities of daily living (ADL) independently [9]. The BI parameters include feeding, bathing, grooming, dressing, bowel control, bladder control, toilet use, transfers (bed to chair and back), mobility of level surfaces and stairs [9]. An assessment score of 100 points indicated that the patient could function independently, 61–99 points indicated mild dependence, 41–60 points indicated moderate dependence, while ≤40 points indicated severe dependence. Evaluations were performed at the time of admission, within 24 h after surgery, and before discharge. In the ERAS group, the schedule patients discharged from the hospital were on postoperative Day 2 depending the patients’ abilities to settle hospital bill quickly.

On postoperative Day 1, the medical staff informed the patients and their families of the discharge plan, as well as carry out relevant discharge guidance which includes home care and schedule follow-ups. Contact numbers were provided should in case the patient encounters any unforeseen eventualities. The duration of hospital stay and the total cost of treatment of all the patients were retrieved from the patients’ records after discharge. All patients were followed up closely on outpatient basis with the aim of identifying any long-term complications using MRI as well as CT scans (the data are beyond the scope of this study and thus not included). However, none of patients reported any long-term complications.

### 2.8 Compliance with ethical standards

All the patients, and their relatives were properly informed about our aim to include them in a study and they freely consented to the use of their documented information. Also, written consents for publication were signed by all the patients included in the study. The hospital also permitted the use of their information for publication. All methods were performed in accordance with the relevant guidelines and regulations. All team members who carried out the ERAS principles were familiar and very motivated to carry out the program. They all strictly avoided the traditional theories, education, as well as attitude toward perioperative care of patients.

### 2.9 Statistical analysis

SPSS 25.0 statistical software was used for data analysis. Mean ± standard deviation was used for the normal distribution of the continuous data, and the *t*-test of two independent samples was used for comparison between groups, the median (quartile) was used for skewed distribution, and the non-parametric test was used for comparison between groups. The number of cases and percentages were used to statistically describe the numerological data, and the C2 test was used for comparison between groups. *p* < 0.05 was considered statistically significant.


**Informed consent:** Written informed consents were obtained from all the patients included in the study.
**Ethical approval:** This study was approved by the Biomedical Ethics Review Committee of West China Hospital, Sichuan University (2022 Annual Review (1583) No.) and retrospectively registered with the China Clinical Trials Registry on 15-03-2023 with registration number: ChiCTR2300069421 (https://www.chictr.org.cn/showprojEN.html?proj=192648).

## 3 Results

### 3.1 General information

A total of 174 patients who underwent TEP tumor resection were included in this study. Out of the 174 patients, 86 patients were recruited into the ERAS group while 88 patients were recruited into the non-ERAS group. In terms of age, gender, and tumor type, there was no statistically significant difference between the two groups, as shown in Table 2. We did not experience losses as well as exclusions after randomization.

**Table 2:** General information of the two groups of patients

Variables	ERAS group (*n* = 86)	Non-ERAS group (*n* = 88)	*χ* ^2^/*t*	*p*-value
**Gender**				
Male	42	37	0.809	0.368
Female	44	51		
**Age**	43.8 ± 11.79	44.2 ± 10.79	0.825	0.365
**Type of tumor**				
Non-functional	60	58	9.131	0.331
Gonadotropin type	12	18		
Pituitary schwannoma	1	0		
Mesenchymal tumors	1	0		
Craniopharyngioma	2	4		
Growth hormone tumors	8	6		
Thyroid-stimulating hormone adenoma	2	0		
Adrenotropic adenoma	0	2		

### 3.2 Self-care ability

The summary of overall self-care ability between the two groups using the BI rating scale is shown in Table 3. There was no statistical difference in the overall self-care ability between the two groups at admission (*p* > 0.05). The overall self-care ability of the ERAS group was higher than that of the non-ERAS group 24 h after surgery (35 points vs 20 points, *p* < 0.001). Also, there was no statistical difference between the overall self-care ability of the ERAS group and the non-ERAS group at discharge (80 points vs 75 points, *p* = 1.135), as shown in Table 3.

**Table 3:** Comparison of overall self-care ability between the two groups [M (P25, P75)] using the BI rating scale assessment

Group	Total	Admission (points)	24 h after surgery (points)	Discharge (points)
ERAS group	86	100 (100, 100)	35 (30, 45)	80 (75, 85)
Non-ERAS group	88	100 (100, 100)	20 (15,20)	75 (70, 85)
*z*-value		−0.828	−10.398	−1.496
*p*-value		0.407	<0.001**	0.135

^∗∗^ *P* < 0.01.

### 3.3 Postoperative urinary catheter

In the ERAS group, urethral catheters were removed under general anesthesia in the anesthesia recovery room before the patient recovered from anesthesia. After catheter removal, only two patients in the ERAS group had urinary catheter-related problems such as benign prostatic hyperplasia (BPH) on admission and their catheter were replaced until discharge, while two patients in the non-ERAS group had urinary problem such as BPH on admission (Table 4). In the non-ERAS group, 6 (6.82%) patients had their catheters removed on the second day after surgery, 46 (52.27%) on the third day, while 36 (40.91%) were removed on the fourth day after surgery. Table 4 shows that there was no significant difference in urinary catheter problems between the two groups (2.32% vs 2.27%, *p* > 0.9999).

**Table 4:** Comparison of postoperative-related catheter problems between the two groups. BPH did not influence urine control after early urethral catheters’ removal. Fisher’s exact test was used

Group	Total	Number of cases (*n*)	Percentage
ERAS group	86	2	2.32
Non-ERAS group	88	2	2.27
*p*-value	>0.9999		

### 3.4 Food intake on postoperative Day 1

Food intake on the first day after surgery in the ERAS group was significantly better than that in the non-ERAS group. About 66% of the patients in the ERAS group could restore 2/3 or more of the food intake, while only 18.2% of the patients in the non-ERAS group could do so. Also, there was a statistically significant difference (*p* < 0.001) on quantity of food intake on the first day after operation as shown in Table 5.

**Table 5:** Comparison of food intake on the first day after operation between the two groups [*n*(%)]

Group	Total	Not eating at all	1/3	1/2	2/3	Full recovery
ERAS group	86	0(0)	13(15.1)	16(18.6)	30(34.9)	27(31.4)
Non-ERAS group	88	11(12.5)	36(40.9)	25(28.4)	16(18.2)	0(0)
*χ* ^2^	55.017					
*p*-value	<0.001^**^					

^∗∗^ *P* < 0.01.

### 3.5 Postoperative complications

There was no significant difference in the overall postoperative complication rate between the ERAS group and the non-ERAS group (22% vs 22.7%, *p* = 0.920). Also, there was no increase in complication rate between the two groups, as shown in Table 6.

**Table 6:** Comparison of postoperative complication rates between the two groups [*n*(%)]

Group	Total	Diabetes insipidus	Electrolyte disturbance	CSF rhinorrhea	Vision loss	Intracranial infection	Total
ERAS group	86	8 (9.3)	7 (8.1)	2 (2.3)	2 (2.3)	0 (0)	19 (22)
Non-ERAS group	88	10 (11.4)	3 (3.4)	3 (3.4)	4 (4.5)	0 (0)	20 (22.7)
*χ* ^2^	0.010						
*p*-value	0.920						

### 3.6 Average length of stay and hospitalization cost

Interestingly, there was statistical significance in the average LOS in the ERAS group compared to the non-ERAS group (4 days vs 7 days, *p* < 0.01) as shown in Table 7. Also, there was statistical significance in the total hospitalization cost in the ERAS group compared to the non-ERAS group (32,886 RMB vs 48,125 RMB, *p* < 0.001) as shown in Table 7.

**Table 7:** Comparison of average LOS and hospitalization costs between the two groups

Comparison of average LOS between the two groups				
Group	Total	M(P25, P75)	*z*-value	*p*-value
ERAS group	86	4(3, 4)	−10.783	<0.001^**^
Non-ERAS group	88	7(6, 7)		

Comparison of hospitalization costs between the two groups				
Group	Total	M(P25, P75)	*z*-value	*p*-value
ERAS group	86	32,886(3,422, 36,232)	−10.993	<0.001^**^
Non-ERAS group	88	48,125(4,614, 51,749)		

^∗∗^ *P* < 0.01.

## 4 Discussion

ERAS nursing approach is specifically the application of the concept of ERAS in nursing, and is an evidence-based perioperative optimization nursing strategy [10]. ERAS strategy endeavors to modify the physiological as well as psychological responses to major surgery [11]. The fundamental principles of the ERAS protocol include preoperative psychotherapy, preoperative nutrition, refrainment of perioperative fasting, as well as carbohydrate intake up to 2 h preoperatively [12]. It also includes standardized anesthetic as well as analgesic regimens such as epidural and non-opioid analgesia as well as early mobilization [12].

This study revealed that the ERAS nursing strategy enhanced patient’s self-care ability within 24 h after surgery, postoperative food intake, average LOS, as well as total hospital cost following TEP tumor resection. However, there was no significant difference in the overall postoperative complication rate between ERAS group and non-ERAS group. Also, in terms of age, gender, and tumor type, there was no statistically significant difference between the two groups.

The BI was originally described by Dr Florence Mahoney and Dorothea Barthel in 1955 [9]. It is a 1–10-item measure of ADL. The BI parameters include feeding, bathing, grooming, dressing, bowel control, bladder control, toilet use, transfers (bed to chair and back), mobility of level surfaces and stairs [9]. Notably, reliability is only one of the critical clinimetric properties to be well thought out when selecting a functional outcome measure. A systematic review and meta-analysis conducted by Duffy et al. revealed excellent interobserver reliability of the standard BI as a stroke outcome measure [13]. Also, the reliability of BI is often seen as a precise strength of this outcome measure for usage as a stroke trial end point [14]. In this study, the BI was very interobserver reliable outcome measure for ERAS nursing of patients undergoing TEP tumor resection just as was done in similar studies [15].

Shortening the time for *in situ* urinary catheter usage after surgery reduced patients’ fear of tubing, increase the time and frequency of early bed mobilization, and promote early recovery. It was observed that patients who underwent pituitary tumor surgery could get out of bed on the first day after surgery if their vital signs were stable to promote the recovery of their gastrointestinal function and improve their quality of life [7,16]. Moreover, urethral catheters were removed before the patients recovered completely from general anesthesia and ECG monitors were stopped early in the ERAS group. This greatly shortened the postoperative monitoring time, created favorable conditions for early mobilization of the patients, improved the comfort of the patients, and promoted the recovery of the patients. We did not observe any significant difference in urethral catheters associated problems such as BPH in both groups.

Our results show that the ERAS nursing strategy can effectively promote the recovery of patients’ self-care ability as early as 24 h after surgery. However, it is worth noting that the self-care ability of the two groups at discharge was not statistically significant, and they were all mildly dependent. Thus, the ERAS nursing care strategy can promote the patients’ early ability to take care of themselves. Also, early stoppage of ECG monitoring as well as removal of urinary catheter and other cannulas boosted mobilization of patients in and out of bed, augmented patients’ comfort, reduced psychological pressure, and all together promoted the restoration of gastrointestinal function thereby promoting the patients’ appetite and accelerating the recovery of food intake.

Postoperative feeding can timely supplement nutrition, correct water and electrolyte balance as well as nitrogen balance, provide body energy, maintain a stable internal environment, and thus facilitate the patient’s recovery [17,18]. This study showed that postoperative feeding in the ERAS group was significantly better than that of the conventional group. Thus, the recovery of self-care ability promotes the early recovery of the patient’s postoperative food intake in 24 h after surgery.

Also, ERAS nursing strategy has shown to decrease complications, improvement in cardiopulmonary function, earlier return of bowel function, as well as earlier resumption of normal activities [19,20]. In this study, postoperative complications evaluated included diabetes insipidus, CSF leakage, intracranial infection, electrolyte imbalance, as well as visual impairment. We did not obverse statistical difference in the incidence of postoperative complications between the ERAS and the control group. Thus, ERAS nursing strategy did not increase postoperative complication rates. It is worth noting that, most postoperative complications usually arise as a result of the surgical technique and the experience of the surgeon [21–23].

An earlier study observed that overall surgical complication rate associated with TEP tumor resection is about 9.1% [21]. The most frequent complications comprised CSF leakage, diabetes insipidus, meningitis, visual deterioration, electrolyte disturbances, hydrocephalus, nausea and vomiting as well as prolonged ventilation [21–23]. However, mechanical injury to the optic nerves as well as chiasm which are caused by trauma often lead to hemorrhage or ischemia resulting in neurological deterioration [21]. Additionally, pressure on cranial nerves, vascular occlusion, or pressure on hypothalamus or brainstem leading to neurological deterioration has also been associated with TEP tumor resection [21].

We observed statistical significance in the average LOS in the ERAS group compared to the control group. Also, there was statistical significance in the total hospitalization cost in the ERAS group compared to the control group. Furthermore, the average LOS was 4 days in the ERAS group compared to 7 days in the control group. The average LOS in the ERAS group was compromised by patients’ inability to settle their hospital bills in time. Also, average LOS for TEP tumor resection is reported to be 3–4 days [7,24,25]. However, some hospitals are discharging selected patients on postoperative Day 1 [26,27]. Furthermore, decreasing LOS is advantageous to both patients as well as the healthcare systems by providing an opportunity to enhance patient experience, improve patient flow, as well as decrease costs. In this study, the ERAS care strategy reduced LOS and hospital costs.

## 5 Limitations

The ERAS protocol, while effective for many surgical patients, has limitations for those undergoing TEP tumor resection. This study was conducted in single center and relatively small sample size for final clinical decision making although our sample size for this study is adequate. Thus, further research is needed to determine the best practices of ERAS nursing strategy for patients undergoing TEP tumor resection. Additionally, the protocol may not address the unique challenges and complications that can arise from pituitary tumor surgery. It is important for healthcare providers to carefully consider the individual needs of each patient and tailor their approach accordingly. Notably, while the ERAS protocol can provide a helpful framework, it should not be considered a one-size-fits-all solution for patients undergoing TEP tumor resection.

## 6 Conclusion

ERAS nursing strategy for patients undergoing TEP tumor resection has significant effects in promoting the recovery of patients’ self-care ability such as urine control and promoted patients’ early feeding. It also shortened patients’ average hospitalization days, and reducing patients’ hospitalization costs, without increasing the incidence of complications. Thus, ERAS nursing strategy for patients undergoing TEP tumor resection is worthy further promotion and application in clinical practice.

## Abbreviations

BPH: benign prostatic hyperplasia
CSF: cerebrospinal fluid
CT: computed tomographic
ECG: electrocardiogram
ERAS: enhanced recovery after surgery
IV: intravenous
LOS: length of hospital stay
MRI: magnetic resonance imaging
TEP: transsphenoidal endoscopic pituitary

## Supplementary Material

Supplementary Table
